# On General and Local Acidity

**Published:** 1879-06

**Authors:** John C. Peters


### ARTICLE IV.

### *On General and Local Acidity.*

BY JOHN C. PETERS, M. D.

Numerous acids exist normally, or are produced in excess in the human frame.

Among the fatty acids, Glycero-phosphoric acid is found in the yolk of the ovum, in the cerebral substance, and in the bile.

*Formic acid* occurs in the fluids with which the muscles, brain and spleen are saturated; also in the sweat in considerable quantities; and in the blood of dogs after a prolonged sugar diet. It is also found pathologically in the blood, in rheumatism, suppression of perspiration, &c.

*Acetic acid* is a constituent of the juices of the muscles and spleen, and has been observed in the perspiration. It is known as one of the ingredients of the gastric juice, and probably occurs in the fluids of the brain. It appears as an occasional constituent of the blood after partaking of spirits in excess.

Secretions both of an acid and akaline character are normal products of the mucous membrane of the stomach ; the former being furnished by the glands of the fundus and body of the organ, which are lined by spheroidal epithelium, and which furnish the true gastric juice ; the latter by those of the pyloric region, whose epithelium is columnar and whose products are those of ordinary mucus, having little or no digestive properties.

The presence of an excessive amount of free acid in the stomach is not in the majority of cases, due to excessive secretion from its coats ; for gastric juice is not only secreted in the presence of food, but depends, with great frequency, on unnatural changes in the food, generally of a fermentative character. Acidity from fermentation may arise (1,) in all cases where digestion is delayed from imperfect supplies of gastric juice ; (2,) when food in a state of fermentation is introduced into the stomach in quantities sufficient to neutralize the antiseptic action of the gastric juice ; (3,) when an excess of mucus possessing a catalytic action is secreted by the stomach ; (4,) when proper changes are not effected during mastication in the amylaceous portions of the food by the salivary and buccal secretions; or, (5,) when an excess of starchy or amylaceous substances is taken with the food.

The results of this fermentative action is shown in the formation of acid products usually derived from the starchy and saccharine elements. The acids so produced, and which are often formed with great rapidity, are, chiefly, the lactic, butyric and acetic, which may appear in great abundance. Gas, which consists principally of carbonic acid and volatile carburetted hydrogen, is apt to appear ; while sulphuretted compounds only appear in the stomach when articles containing this element have been taken with the food.

Fœrichs divides the fermentative processes which may take place in the stomach into the following : (a,) Alcoholic ; (b,) Lactic ; (c,) Butyic. The lactic, which is a simple acid fermentation, causes no evolution of gas. The butyric succeeds the lactic and is associated only with the formation of carbonic acid ; but as butyric acid is volatile, its taste is perceptible in the eructations. Alcoholic fermentation may be attended by the formation of acetic acid and give rise to acid eructation. The conditions of fermentation are delay of absorption and the presence of much mucus in the stomach.

*Butyric acid* appears in flesh and the juices of the spleen ; also in milk, sweet and secretions of the sebaceous follicles of many parts of the body, as the armpits and genitals. It is also found, as before said, in the stomach and bowels, as a product of the fermentation of hydro-carbons.

*Palmitic acid* is an element in almost all fats of the vegetable and animal kingdom. It forms with glycerin, a compound occurring naturally and abundantly in human fat.

*Stearic acid* is also a widely spread constituent of the animal neutral fats in the human body, but is exceeded in quantity by palmitic acid.

*Oleic acid* is scentless and tasteless, and is found as a most important constituent of the neutral fats of the body.

*Margaric acid* was formerly believed to be the most widely distributed of all the animal fats ; but a mixture of equal parts of palmitic and stearic acids has naturally the same composition as margaric acid.

*Lactic acid* is a decomposition product, easily formed from starch or sugary fluids by fermentation, and is found in the stomach. It can also be formed from Inosite or muscle-sugar, and is supposed to play a large part in the rheumatic diathesis. It is also found in the intestinal canal as a decomposition product of ingested carbo-hydrates ; and if it is the acid of gastric juice, intestinal digestion may include a larger role than is usually admitted. It has also been found in the brain and various gland-fluids of the body

Frey says there can be no doubt, when it is not a product of fermentation, it is derived from the decomposition of histogenic substances.

*Hydrochloric acid* is only found free in the gastric juices. The presence of an acid is necessary for the transformation which essentially constitutes the digestive function of the stomach. In the gastric juice, therefore, pepsin is united with an acid in a free state, the exact nature of which has been much disputed. Some maintain that it is hydrochloric acid; others, *phosphoric acid* or acid phosphate of lime; and others still, lactic acid. Kuss says the latter opinion is now most generally held, but the presence of muriatic acid in excess in the stomach is absolutely necessary to retain the chloride of sodium in the blood.

The real gastric juice is only secreted when an albuminoid substance like meat, fibrin albumen, &c., or an aliment which essentially requires the action of gastric juice to digest it, is put into the stomach.

*Oxalic acid* is found abundantly throughout the vegetable kingdom, and appears as an end-product in the oxidation of most animal and vegetable substances. With one atom of calcium, it forms the neutral oxalate of calcium; which is almost the only one of its salts found in the human body. Still, a large amount of it is never met with in the body; although in very minute quantity, it is a normal constituent of the urine. It is most frequently observed after a vegetable diet, and the use of drinks containing a large amount of carbonic acid. It appears also in conjunction with disturbance of the respiratory functions, and may give rise to mulberry calculus. It has been met with in the gall bladder, and in uterine mucus. It may arise in excess from a preponderating use of vegetable food, and from the decomposition of various animal matters; especially from the oxidation of uric acid, as after the injection of urates into the blood, the amount of both urea and oxalic acid is increased in the urine. The oxalic acid diathesis will be treated of hereafter.

*Succinic acid* arises from the oxidation of fatty acids, and from the fermentation of different organic acids. It was formerly supposed only to occur as a pathological constituent of the body in encysted tumors and dropsical fluids; but it has been found in a number of gland-juices, viz., those of the spleen, thymus and thyroid. It also exists in the blood of herbivorous mammals, in human urine and that of carnivora and vegetable feeders, after fatty diet or the use of malic acid.

The nitrogenous animal-acids are of course not found in the vegetable kingdom at all; they are all mutation products of histogenic matters, or plastic alimentary materials, and all except two are constituents, either of the urine or the bile, forming essential elements of these secretions.

*Inosinic acid* is a constituent of muscle-juice, and probably produced from fleshy fibre. Hydrotinic acid is a constituent of human sweat, and probably derived from the above. It is not known what disorders of the muscular system their excess may occasion.

*Uric acid* is a derivative of ammonia, and when deposited in the urine is discolored by the pigmentary matters of that fluid, so that its crystals appear yellowish or brownish. Dumb-bell crystals may appear as a natural product, or may be produced by the decomposition of urate of potassium. It requires 14,000 parts of cold and 1,800 of hot water to dissolve it. It is the source of urea, allantoin, oxalic acid and glycin; and is a constant constituent of human urine in very minute quantities, however, only one part per thousand consisting of it. Its amount varies but little according to the nature of the food taken, but much in certain morbid conditions, especially in gout. It has also been found in the blood, in the fluids of the brain, lungs and spleen. It is a mutation-product of nitrogenized tissue constituents.

*Hippuric acid* is a glycin, or amido-acetic acid, in which one atom of hydrogen is replaced by benzoyl, the radicle of benzoic acid. On being heated with acids and alkalies, it is split up into benzoic acid and glycin, after taking up water. It appears in human urine in about the same amount as uric acid, but in longer amount in certain diseased states of the system. It has not been found in the blood or juices of the system, but in the scales of ichthyosis. Frey says it is a fact of great interest that benzoic, kinic and cinamic acids, oil of bitter almonds and of tolu, when introduced into the stomach, are excreted as hippuric acid through the kidneys. On the oxidation of protein substances by permanganate potash, a great quantity of benzoic acid is developed.

*Glycocholate acid* which belongs to the bile, is analogous in composition to hippuric acid, splitting up like the latter into glycin, and a non-nitrogenous acid, viz., cholic acid.

*Glycocholic of sodium* is an essential constituent of bile.

*Taurocholic acid* splits up, not into glycin, but into cholic acid and taurin, an indifferent substance containing sulphur. It dissolves fats, fatty acids, and choles-tearin, with great readiness. Combined with sodium it forms the second chief constituent of the bile of man and numerous mammels, viz., the tauro-cholate of sodium.

*Lencin*, or amidocapronic acid, or starch-capronic acid, the latter occurring in a free state with glycerin in butter and sweat. It can be produced by the artificial decomposition of protein compounds, glutin substances and elastin, by means of acids and alkalies. It is also a product of the putrefaction of protein substances, especially tyrosin. It is a physiological decomposition product of great interest, as it is widely distributed throughout the body. We must hence distinguish between lencin produced by the putrefaction of histogenic substances, and that formed physiologically in the living body. The latter form is generally accompanied by tyrosin, which is a constituent of many organic fluids and gland juices, and under some diseased conditions is often unusually abundant in organs where scarcely a trace can be detected in health.

It occurs normally in the spleen, pancreas and its secretions, the salivary glands and saliva, in the lymphatic, thymus and thyroid glands and in the fluid saturating the pulmonary tissue. In the healthy liver and brain it is scarely to be found; and it is rare in the muscles except those of the heart where it is not unfrequently seen as a pathological product. At times it is present in large quantities in the kidneys and may pass into the urine.

These facts prove the existence in different organs of peculiar and distinct series of mutations among their histogenic substances. Thus lencin is no mutation product of muscle, except, perhaps, in fatty hearts, but is of many grandular structures. It is formed physiologically from many protein compounds, gelatin yielding substances and elastin; it can also be formed from albuminates by the action of one of the ferments existing in the pancreatic juice. It is partially excreted with the glandular secretions and appears in the intestinal canal. In the lymphatic and blood vascular glands it is found in conjunction with ammonia, suggesting the supposition that lencin may be resolved there into ammonia and volatile fatty acids, which it certainly does in the lower part of the intestinal canal. It will be further treated of under Leucaemia.

*Tyrosin* is also an amido, or starch-acid; it arises like Lencin from the decomposition of protein matters, but not from elastic and gelatin. Keratin and animal mucus also yield more tyrosin far than the original protein matters. It is a physiological companion of Lencin both in the normal and diseased body, but does not occur so extensively. Like lencin it occurs not in the healthy liver, probably because it then undergoes rapid transformation into other compounds; but is found in diseased livers. It has been found alone in no inconsiderable amount in the spleen and tissue of the pancreas, and in the digestion of albumen in pancreatic juice.

*Glycin*, or glycocoll, or glutin-sugar, is in reality an amido acetic acid, which does not occur free in the system but may arise from the splitting up of hippuric-uric, and one of the biliary acids, viz.: the glycocholic, and from the decomposition of glutin and chondrin. Some unknown substance, nearly related to glycin, is probably formed from glutinous matters, and which, in combination with cholic acid, constitutes glycocholic acid; and with benzoic, hippuric acid. It then becomes free in the form of glycin upon the absorption of water and splitting up of the acids. Glycin leaves the body partly through the kidneys with the hippuric acid, and is partly re-absorbed into the blood as a component of glycocholic acid.

*Taurin* is an amido-sulf-ethylemic acid and may be obtained by the splitting up of one of the biliary, viz.: the taurocholic; and contains all the sulphur of the bile. It becomes free on the decomposition of taurocholic acid in the body, and then appears in abnormal as well as putrid bile; and in the lower part of the intestinal canal. It has been found in the juices of the renal and pulmonary tissues and was formerly described as pulmonic acid. The supra-renal capsules contain it, but the blood does not. From the fact that it contains sulphur there can hardly be any doubt that it is derived from albuminous matters. Taurin splits up by the fermentative action of the mucus of the gall bladder, in the presence of alkalies, into carbonate of ammonium, sulphurous and acetic acids. The acetic acid combined with an alkali is changed into a carbonate; and the sulphurous acid in combination with sodium becomes converted, at a later period, into sulphuric acid; so that we meet with Na. 2, C. S. O. 4, in putrefying bile. As most of the bile poured out into the intestines is re-absorbed, the sulphates ultimately leave the body with the urine.

Stomachal acidity can only be palliated, not cured by alkalies. As it arises from a fermentative process, *sulphurous acid*, which arrests putrefaction and fermentation has been suggested. Dr. Lawson speaks highly of it as a remedy for pyrosis, which he says it never fails to relieve, while the sulphites are said to be useless. The sulphites and hypo-sulphites have been used to destroy sarcinae and torlane in the stomach, as these are products or agents of fermentation.

Ringer thinks that alkalies have not only the power to increase the secretion of the gastric juice, which is an acid secretion, but when applied to the orifices of glands with *acid* secretions, increase their secreting power; while, when applied to glands with alkaline secretions, it checks or lessens them.

He repeats that alkalies increase the secretion of the gastric juice, and may thus prove useful in promoting digestion, if given shortly before a meal; while, if given soon after a meal, they will neutralize the acid of the gastric juice, and effectually retard and impede digestion. Schiff, on the other hand, believes that real gastric juice is only secreted when albuminoid aliment is taken into the stomach, when the mucous coat becomes red and turgescent, and then ensues an abundant secretion of gastric juice, which soon transforms the albuminoid aliment into albuminose. Hence the secretion of gastric juice is said to be the result of a special sensibility on the part of the mucous membrane of the stomach, which cannot be deceived even by alkalies; but the presence of an aliment suited to digestion by means of the gastric juice is always needed to produce it. Instead of alkalies, Schiff gives soup an hour or two before meals, which causes the stomach to secrete a purely acid fluid, which serves to dissolve the peptogenous elements, and these becoming absorbed and mixing with the blood, enable it to furnish pepsin to the glands of the stomach, when the secretion of gastric juice becomes constantly more active, or in short, *peptic*, and thus prevents the fermentation of the coming meal.

When, on the other hand, a patient complains of heartburn and acid eructations, these may be removed at once by the exhibition of a bicarbonate alkali, which neutralizes the excess of acid in the stomach. But unless combined or preceded by a bitter tonic, this treatment will generally prove merely palliative.

The milder alkalies, as the bicarbonates of potash, soda, or magnesia, are used with great benefit in diarrhœa, due to excess of acids in the intestines. By neutralizing the excess of acid they arrest the diarrhœa.

As alkalies pass readily into the blood, the alkalinity of this fluid must be increased by them. They have also been given for excess of uric acid in the urine, with the expectation of oxidizing this product of the nitrogenous tissues, and so converting it into urea. They do not oxidize the uric acid in the blood, but by rendering the urine weakly, acid or even alkaline, they convert the excess of uric acid into a more soluble urate. This treatment will also prevent the growth of uric acid calculi. Painful micturition not unfrequently depends on the existence of uric acid or bi-urates in the form of speiular crystals, which in their passage irritate the pelves of the kidneys and the ureters almost as severely as calculi, and also the urethra. Alkalies dissolve these, and render them innocuous.

The bicarbonate or citrate of potash, or Rochelle salts, are often employed in rheumatism, which is supposed to be caused by an excessive formation of lactic acid in the body. In many cases the rheumatic pain is much relieved as soon as the patient is well under the action of the alkali, and the urine has become fairly alkaline.

Renal calculi are generally composed of uric acid only, and if the urine be kept alkaline, it will in time reduce the calculus sufficiently to allow it to pass down the ureter. Ringer says we certainly meet with patients complaining of much pain in the back, passing bloody urine containing a large quantity of uric acid crystals and a little pus, who are curable with large doses of citrate of potash.

Gout stones are composed of the urates, and as the urate of lithia is the most soluble of the uric acid salts, a strong solution of lithia is applied locally to remove gouty enlargements and ulcers. In the latter case the urates being intimately mixed with the connective tissue, and oozing very slowly through the broken surface, are dissolved and washed away by the lithia solutions, thus enabling the sore to heal. The citrate of lithia is to be preferred, but a strong solution of citrate of potash is said by Ringer to be nearly, if not quite as useful. It probably converts the bi-urates into neutral urates, and in the more soluble form they are carried off through the skin. Equal parts of citrate of potash and water may be used, as neither it nor the citrate of potash irritate the skin.

Mr. Mialhe has pointed out that the principal animal fluids, with the exception of the urine and sweat, are alkaline; but owing to sedentary occupations and animal diet, excretion is very imperfectly performed, and a great deal of acid which ought to be elminated, is retained within the system, and a very large quantity of alkaline ingesta is required to neutralize the retained acids. On the other hand, an active and a laborious life, and a mostly vegetable diet, render the toleration of alkalies more difficult. The treatment must vary as the fatty acids, nitrogenous animal acids, and the non-nitrogenous or vegetable acids abound.

Stille prefers *soda* in those forms of dyspepsia in which acidity of the primæ viæ is the most striking symptoms; whether the acidity arises from acetous fermentation of amylaceous or saccharine food, the ingestion of sour substances, the development of carbonic acid gas in the bowels, an excessive formation of fatty acids from the use of too much oleaginous food, and a superformation of fatty acids in the system, especially when there are sour vomitings, eructations, and dejections, with the tongue clear, red and even shining, and the mouth dry. Also in those numerous ailments of young children which arise from the ingestion of sour milk, or the generation of acid in the stomach and bowels, marked by colic and the dejection of frequent sour and greenish stools; or of a thin liquid containing small fragments of fæces resembling portions of hard boiled eggs, or of spinach and eggs finely hashed together, &c. Trousseau succeeded in diminishing the mortality among young children in the Neckar hospital, by adding ten grains of bicarbonate of soda to each quart of milk given them.

*Magnesia* is also advised by Stille in most forms of derangement of digestion, attended with acidity of the stomach. It has even arrested the vomiting of pregnancy when the rejected fluid was strongly acid. In all cases of sour stomach, heart-burn, colic, sick headache and mental depression, especially when accompanied by constipation.

*Bismuth* is an astringent and sedative, very useful in the various forms of pyrosis, whether acid, alkaline or neutral. Graves successfully treated acidity of the stomach with it, but he generally mixed it with magnesia. Flatulent dyspepsia from acid fermentation yields more or less to it; but it may be mixed with equal quantities of vegetable charcoal.

*Acids* are astringents which check profuse secretions, and Ringer says that repeated and careful experiments have established the fact that dilute acids taken into the stomach check its secretions; and that practical men well know that acids check acidity, removing the acid eructations, the heart-burn and sense of discomfort at the chest and hypogastrium arising from excess of acid in the stomach. Hydrochloric or nitric acid is generally preferred. Dr. Day, of St. Andrews, noticed in patients greatly annoyed by eructations of offensive gas with the odor and flavor of rotten eggs, consisting largely of sulphuretted hydrogen gas, were apt to have their urine loaded with oxalic acid, for which he strongly recommends the employment of the mineral acids. Nitric acid is also of great service in the treatment of dyspeptics with oxalic acid in their urine, but who are free from sulphuretted hydrogen eructations and who suffer from great mental depression.

In another class of cases, Ringer says, not unfrequently, soon after a meal, a fluid regurgitates almost unconsciously into the mouth so strongly acid that it sets the teeth on edge. The exhibition of nitric or hydrochloric acid, shortly before each meal, in virtue of its astringency, almost immediately removes this acid pyrosis. He continues that it need hardly be said that acids given soon after a meal to patients troubled with acidity and heart-burn greatly aggravates their sufferings. This is adding fuel to the fire.

*Ammonia carb*, in doses of five to seven grains in some aromatic water, is very useful in acidity of the primæ viæ, heart-burn and flatulence, particularly when occuring in cases of atonic dyspepsia, or in hysterical females, in which the ordinary alkalies are too debilitating. The aromatic spirit of ammonia should also be thought of.

*Liquor calcis* is thought particularly useful in cardialgia and dyspepsia arising from acidity, especially in persons whose urine shows a strong acid reaction and when vomiting is a prominent symptom. It is best given in milk in doses of ℥ ss. to ℥ ij. It is often permanently and speedily effectual.

*Nux vomica*, in doses of five drops before each meal, is said by Ringer to be of great service in a group of symptoms, including weight at the pit of the stomach after food, acidity, heart-burn and flatulence, accompanied by heat and weight at the top of the head. It acts as a tonic to the muscular coat of the stomach, and to the vaso-motor nerves, contracting the capillaries and thus removing congestion of the stomach and brain. Ergot may be used for the same purpose.

Ringer says few remedies are more useful in certain diseases of the stomach than *Arsenic.* When the vomiting is intensely bitter and sour, and of a green color, it will not only arrest the vomiting, but will often improve the state of the stomach and restore both appetite and digestion. It sometimes removes heart-burn.

The liquor Ammoniæ Anisatus of the Germans is a useful and pleasant preparation; often more so than aromatic spirits of ammonia.—*Physician and Pharmacist.*

